# Complement proteins C1q, C3, and Factor H are associated with early tau pathology and synaptic integrity in persons with pre‐symptomatic Alzheimer’s disease

**DOI:** 10.1002/alz.088297

**Published:** 2025-01-09

**Authors:** Julia Loncke, Cynthia Picard, Anne Labonté, Henrik Zetterberg, Kaj Blennow, Ann Brinkmalm, John C.S. Breitner, Judes Poirier, The PREVENT‐AD Research Group

**Affiliations:** ^1^ Integrated Program in Neuroscience, Faculty of Medicine and Health Science, McGill University, Montréal, QC Canada; ^2^ Department of Psychiatry, McGill University, Montréal, QC Canada; ^3^ Douglas Mental Health Research Centre, Montreal, QC Canada; ^4^ Centre for Studies on Prevention of Alzheimer's Disease (StoP‐AD Centre), Montréal, QC Canada; ^5^ Douglas Mental Health University Institute, Montréal, QC Canada; ^6^ Centre for Studies on Prevention of Alzheimer's Disease (StoP‐AD Centre), Douglas Mental Health University Institute, Montréal, QC Canada; ^7^ Department of Psychiatry and Neurochemistry, Institute of Neuroscience and Physiology, The Sahlgrenska Academy, University of Gothenburg, Mölndal, Gothenburg Sweden; ^8^ Department of Psychiatry and Neurochemistry, Institute of Neuroscience and Physiology, The Sahlgrenska Academy at the University of Gothenburg, Mölndal Sweden; ^9^ Department of Neurodegenerative Disease, UCL Queen Square Institute of Neurology, University College London, London, ‐ United Kingdom; ^10^ UK Dementia Research Institute at UCL, London United Kingdom; ^11^ Hong Kong Center for Neurodegenerative Diseases, Hong Kong China; ^12^ Wisconsin Alzheimer's Disease Research Center, University of Wisconsin School of Medicine and Public Health, Madison, WI USA; ^13^ University of Science and Technology of China, Hefei, Anhui China; ^14^ Institute of Neuroscience and Physiology Sahlgrenska Academy at the University of Gothenburg, Gothenburg Sweden; ^15^ Clinical Neurochemistry Laboratory Sahlgrenska University Hospital, Mölndal Sweden; ^16^ Department of Neurodegenerative Disease, UCL Queen Square Institute of Neurology, University College London, London United Kingdom; ^17^ Paris Brain Institute, ICM, Pitié‐Salpêtrière Hospital, Sorbonne University, Paris France; ^18^ Clinical Neurochemistry Laboratory, Sahlgrenska University Hospital, Mölndal Sweden; ^19^ Department of Medicine, McGill University, Montréal, QC Canada

## Abstract

**Background:**

The immune complement system is key to the elimination of redundant neural connections in the brain through a process called synaptic pruning. In neurodegenerative diseases such as Alzheimer's disease (AD), this system may result in excessive synapse loss, leading to brain atrophy and cognitive impairment. While increased cerebrospinal fluid (CSF) levels of complement proteins have been observed in patients with AD dementia, no studies have yet investigated the role of complement in the pre‐symptomatic phase of AD, nor throughout its progression.

**Method:**

We used Luminex technology to assess complement protein levels in 566 CSF samples obtained over five years from 160 high‐risk asymptomatic subjects with familial history of AD [the PREVENT‐AD cohort; Breitner et al, 2016: JPAD 3,(4) 236]. Baseline complement levels were contrasted with CSF biomarkers of AD pathology (Aβ42, p(181) and total tau, NFL) and markers of synaptic dysfunction (GAP‐43, SYT1, SNAP‐25, ADAM22, ADAM23). Analyses were stratified by sex and ApoE4 carrier status. Currently pending are additional longitudinal analyses probing complement levels versus PET‐quantified cerebral amyloid and tau deposition, MRI volume of AD‐implicated structures, and cognitive ability (RBANS).

**Result:**

Preliminary analyses revealed significant positive associations between complement proteins and p‐(181)tau (C1q: R^2^ = .238, p < .0001; C3: R^2^ = .041, p = .0099; C3b: R^2^ = .027, p = .0384; Factor H: R^2^ = .128, p < .0001) and total tau levels (C1q: R^2^ = .247, p < .0001; C3: R^2^ = .023, p = .0543; Factor H: R^2^ = .128, p < .0001). Complement relationships with synaptic protein levels were more significant overall in females, which showed the following associations [GAP‐43 (C1q: R^2^ = .389, p < .0001; Factor H: R^2^ = .315, p = .0002); SNAP‐25 (C1q: R^2^ = .367, p < .0001; C3: R^2^ = .106, p = .0459; Factor H: R^2^ = .278, p = .0072); SYT1 (C1q: R^2^ = .316, p = .0002; Factor H: R^2^ = .246, p = .0015)].

**Conclusion:**

There is a clear association between complement levels, tau pathology, and synaptic markers in the asymptomatic phase of AD, especially in women. Longitudinal and multimodal investigation is ongoing to further characterize these relationships.

